# Granulocyte macrophage-colony stimulating factor: A key modulator of renal mononuclear phagocyte plasticity

**DOI:** 10.1016/j.imbio.2018.10.007

**Published:** 2019-01

**Authors:** Katie J. Mylonas, Jennifer Anderson, Tara A. Sheldrake, Emily E. Hesketh, James A. Richards, David A. Ferenbach, David C. Kluth, John Savill, Jeremy Hughes

**Affiliations:** The University of Edinburgh/ Centre for Inflammation Research, The Queen's Medical Research Institute, Edinburgh BioQuarter, 47 Little France Crescent Edinburgh EH16 4TJ, Scotland, United Kingdom

**Keywords:** Macrophage, DC, Mononuclear phagocyte, Inflammation, M-CSF, GM-CSF, Mφ, macrophage, DC, dendritic cell, BMMφ, bone marrow-derived macrophages, BMDCs, BM-derived dendritic Cells, M-CSF, macrophage colony stimulating factor, M-CSFR, macrophage colony stimulating factor 1 receptor, GM-CSF, granulocyte macrophage colony-stimulating factor, CCR7, C-C chemokine receptor type 7, UUO, unilateral ureteric obstruction, MLR, mixed leukocyte reaction, FACS, fluorescence associated cell sorting

## Abstract

Macrophage-colony stimulating factor (M-CSF) and granulocyte macrophage-colony stimulating factor (GM-CSF) play key roles in the differentiation of macrophages and dendritic cells (DCs). We examined the effect of treatment with M-CSF-containing macrophage medium or GM-CSF-containing DC medium upon the phenotype of murine bone marrow-derived macrophages and DCs. Culture of macrophages for 5 days in DC medium reduced F4/80 expression and increased CD11c expression with cells effectively stimulating T cell proliferation in a mixed lymphocyte reaction. DC medium treatment of macrophages significantly reduced phagocytosis of both apoptotic cells and latex beads and strongly induced the expression of the chemokine receptor *CCR7* known to be involved in DC trafficking to lymph nodes. Lysates of obstructed murine kidneys expressed both M-CSF and GM-CSF though M-CSF expression was dominant (M-CSF:GM-CSF ratio ∼30:1). However, combination treatment with both M-CSF and GM-CSF (ratio 30:1) indicated that small amounts of GM-CSF skewed macrophages towards a DC–like phenotype. To determine whether macrophage phenotype might be modulated *in vivo* we tracked CD45.1^+^ bone marrow-derived macrophages intravenously administered to CD45.2^+^ mice with unilateral ureteric obstruction. Flow cytometry of enzyme dissociated kidneys harvested 3 days later indicated CD11c and MHC Class II upregulation by adoptively transferred CD45.1^+^ cells with CD45.1^+^ cells evident in draining renal lymph nodes. Our data suggests that GM-CSF modulates mononuclear phagocyte plasticity, which likely promotes resolution of injury and healing in the injured kidney.

## 1 Introduction

Renal mononuclear phagocytes play a key role in innate and adaptive immunity in renal health and disease (reviewed in Nelson et al., 2012) and have been described as either macrophages (Mφ) or dendritic cells (DCs) according to various criteria (Rogers et al., 2014). Mφ have historically been ascribed various roles in phagocytosis, renal inflammation, fibrosis and regeneration (Duffield, 2010; Wang and Harris, 2011; Anders and Ryu, 2011), whereas DCs are thought to be specialized for the capture, processing and presentation of antigen and the subsequent instruction of immune cells with both pathogenic and tolerogenic roles being described (Teteris et al., 2011; Hochheiser et al., 2011). Considerable flexibility, heterogeneity, and complexity is recognized within the myeloid–monocyte developmental lineage (Hume et al., 2002; Mosser and Edwards, 2008). Recent work has shown that renal tissue resident Mφ populations have different developmental origins, with subsets comprised of monocyte-derived cells, but also of embryonic origin (Ginhoux and Guilliams, 2016). In the kidney, mononuclear phagocytes have been found to comprise of various groups based on combinations of cell surface markers (e.g. CD11c, F4/80, CD11b) and phenotype/function (Kawakami et al., 2013; Viehmann et al., 2018; Lee et al., 2018). Kawakami et al reported the presence of five discrete subsets of renal mononuclear phagocyte in the steady state, most of which display phenotypic and functional characteristics of both Mφ and DCs i.e. they are positive for both CD11c and F4/80, and display both phagocytic and antigen presentation abilities (Kawakami et al., 2013).

The discrete growth and differentiation factors macrophage colony-stimulating factor (M-CSF) and granulocyte macrophage colony-stimulating factor (GM-CSF) induce the differentiation of Mφ and DCs from hematopoietic progenitors (Kipari et al., 2006; Inaba et al., 1992). Mφ and DCs may be generated *in vitro* and, although they are unlikely to have an exact counterpart *in vivo*, these cells have been used to dissect various cellular functions of Mφ and DC such as the interaction with apoptotic cells (Lucas et al., 2006). Also, recent work has demonstrated the beneficial effects of using bone marrow-derived Mφ as a substrate for cell therapy in acute kidney injury (Ferenbach et al., 2010), glomerulonephritis (Wilson et al., 2002), and renal fibrosis (Yamagishi et al., 2001). However, it is unclear whether such cells undergo phenotypic changes following localisation in inflamed tissues as a result of exposure to tissue-derived growth and differentiation factors.

In this study, we examined the phenotypic and functional plasticity of murine bone marrow-derived Mφ (BMMφ) and DCs (BMDC) exposed to M-CSF or GM-CSF-containing medium either sequentially or in combination. Key phenotypic readouts included the flow cytometric analysis of cell surface markers (F4/80, CD11c & MHC Class II) and realtime PCR for the chemokine receptor *CCR7* involved in DC egress from tissues to lymphoid organs. Key functional readouts included phagocytosis (Lucas et al., 2006), and stimulation of T cell proliferation in the mixed lymphocyte reaction (MLR). In this study, we determined the kinetics of renal M-CSF and GM-CSF protein expression for the first time during unilateral ureteric obstruction (UUO) and tracked the fate and phenotype of CD45.1^+^ BMMφ adoptively transferred to CD45.2^+^ C57B/6 mice with UUO. M-CSF and GM-CSF are known to be important regulators of mononuclear phagocyte plasticity. This work shows that exposure to even small quantities of GM-CSF in the inflamed kidney promotes the acquisition of features in Mφ more commonly associated with DCs, that likely promote the resolution of inflammation and effective renal repair.

## 2 Materials and methods

### 2.1 Experimental mice

6–10 week old male mice on the C57BL/6 background (CD45.1 or CD45.2) were bred and maintained in conventional barrier unit facilities at the University of Edinburgh or purchased from Harlan, UK. These units are regularly tested in accordance with the Felasa 2014 recommendations, which involves testing for various infectious agents, including parasites.

### 2.2 Ethics statement

All animal work was compliant with IACUC guidelines, conducted in accordance with the UK Government Animals (Scientific Procedures) Act 1986 and was approved by the University of Edinburgh Ethical Review Committee.

### 2.3 Cell culture

Mφ medium consisted of either (i) RPMI (GIBCO, UK) supplemented with 25% FBS (GIBCO), 25% L929 supernatant (a source of M-CSF), 2 mM l-glutamine, 0.25U/ml penicillin and 100 μg/ml streptomycin or (ii) 20 ng/ml recombinant murine (rm) M-CSF (Invitrogen, UK) in complete RPMI (10% FBS, l-glutamine and Penicillin/Streptomycin). DC medium consisted of either (i) 10% GM-CSF conditioned medium in complete RPMI or (ii) 20 ng/ml rm GM-CSF (Invitrogen, UK) added to complete RPMI.

BMMφ and BMDC were prepared from C57BL/6 bone marrow as previously described (Kipari et al., 2006). Cells were plated (7.5 × 10^6^ cells/plate), cultured in Mφ medium with adherent Mφ evident at day 7. BMDC were similarly generated using DC medium with supplementary medium added at days 2 and 4. DCs were present as non-adherent cells at day 7. In some experiments, bone marrow cells were cultured for 7 days in Teflon pots. In medium switching experiments, day 7 adherent Mφ and non-adherent DCs were removed to fresh 6-well culture plates (1–1.5 × 10^6^ cells/ well in 3 ml media) and cultured for a further 5 days in either conventional or recombinant Mφ medium, DC medium or a mix of Mφ/DC media (i.e. Mφ/Mφ, Mφ/DC, DC/DC, DC/Mφ, Mφ/Mix and DC/Mix). Following peritoneal lavage, peritoneal Mφ were purified by adhesion to tissue culture plastic.

### 2.4 Unilateral ureteric obstruction and enzymatic dissociation of organs

UUO was performed in anaesthetized 6–10 week male C57BL/6 mice as previously described (Kipari et al., 2006). The obstructed left kidneys, and livers, were diced into small pieces and incubated in 1.6 mg/ml Collagenase B (Roche, West Sussex, UK) and 100 μg/ml DNAse 1 (Ambion, Warrington, UK) in RPMI medium at 37 °C for 45 min with gentle agitation. Tissue was centrifuged (300 *g* × 5 min) and incubated with 100 μg/ml DNAse 1 in RPMI medium for 15 min at room temperature. Following centrifugation and resuspension in 1 ml RPMI, digested kidney and liver tissue, and spleens were gently pressed through a 40-μm cell strainer using a flattened pestle. Cells were centrifuged (300 *g* × 5 min) and red blood cells lysed with lysis buffer (Sigma) for 5–10 min at room temperature before washing once in PBS. The resultant single cell suspension was then analyzed by flow cytometry.

### 2.5 Flow Cytometry and fluorescence associated cell sorting (FACs)

2.5 × 10^5^ −1 × 10^6^ cells from cell culture or tissue digest, were blocked with 10% mouse serum (20 min on ice) and incubated for 30 min on ice with the fluorochrome conjugated primary antibody diluted in PBS with 10% mouse serum. Antibodies included anti-F4/80-PerCp Cy5.5 (1:50), anti-F4/80-APC (1:100), anti-CD11c-APC (1:100), anti-CD11c-PE (1:100), anti-MHC class II-FITC (1:100), anti-CD11b-APC (1:100) and anti-CD45.1-Pacific Blue (1:100) and appropriate isotype control Abs. The cells were then washed once in PBS, some fixed in 5% formalin before acquisition and analysis (BD FACStation, FacsAria and FlowJo software). Propidium Iodide (1:1000) was added before acquisition to quantify dead cells. CD11b + cells were sorted from naïve and post-UUO kidneys by FACS using the FACsAria II. These cells were frozen at −80 °C in TRIizol Reagent (Invitrogen) before RNA extraction and real-time RT-PCR for CCR7.

### 2.6 Phagocytosis assay

BMMφ underwent various treatments before being incubated with either CM green labeled apoptotic thymocytes or fluorescent beads (10:1 ratio) for 60 min. Cells were vigorously washed and detached from the plates prior to quantification of phagocytosis by flow cytometry.

### 2.7 RNA extraction and real-time RT PCR

RNA was recovered from cells by resuspension in TRIzol reagent (Invitrogen). Total RNA was extracted and approximately 1 μg of RNA used to synthesize cDNA using MMLV reverse transcriptase (Stratagene, UK). Relative quantification of *CCR7* was measured by real-time PCR, using the 7500 Fast Real-Time PCR System (Applied Biosystems) with the expression level normalized to the housekeeping gene β-actin. PCR amplifications were performed in a total volume of 20 μl containing 1 μl cDNA, 4 mM MgCl_2_, 0.3 mM primers and the SYBR Green I mix. Amplifications were performed in the following conditions: 30 s denaturation at 95 °C, 5 s annealing of primers at 55 °C and 12 s elongation at 72 °C, for 40–50 cycles. Primers for PCR analysis were:

β*-Actin*: TGGAATCCTGTGGCATCCATGAAAC, TAAAACGCAGCTCAGTAACAGTCCG.

*CCR7:* TGCTTCAAGAAGGATGTGCGG, GAGGAAAAGGATGTCTGCCACG

### 2.8 Mixed leukocyte reaction

C57BL/6 BMMφ and BMDC treated with Mφ medium or DC medium were co-cultured (1 × 10^5^ cells/well) in 96-well flat-bottomed plates with splenocytes from BALB/c mice. After 48 h, 1μCi of [^3^H]-TdR in 10 μl complete medium was added to each well and plates incubated overnight before harvesting and counting using a liquid scintillation counter (Microbeta 1450, Trilux). Quadruplicate measurements per sample were performed and results expressed as counts per minute.

### 2.9 Fluorescent cell labeling

Cells were labeled with the PKH67 Green Fluorescent Cell Linker Kit for General Cell Membrane Labeling (Sigma) according to the manufacturer’s instructions (Sigma). Briefly, cells were washed in serum free RPMI medium, suspended in Diluent A (Sigma) and then mixed with an equal volume of dye working solution (containing PKH green). After incubation for 2–5 min, FBS was added for one minute before cells were washed 3 times in complete RPMI (10% FBS, pen/strep, l-glut). Labeling efficiency was then assessed by flow cytometry.

### 2.10 M-CSF and GM-CSF protein measurement

Mice underwent UUO and the obstructed kidneys removed on days 3, 5 and 7 (3 mice per time point, 5 experiments performed). 30 mg of snap frozen control or obstructed kidney tissue was placed in 300 μl RIPA buffer (plus protease inhibitors), homogenised using plastic pestles and placed on ice for 10 min. Supernatants were removed following centrifugation (10 min. at 10,000 *g*) and the concentrations of M-CSF and GM-CSF were measured by ELISA (R&D Systems, UK) according to manufacturer’s instructions.

### 2.11 Adoptive transfer of bone marrow-derived macrophages

BMMφ were generated by culture of bone marrow cells from CD45.1^+^ C57BL/6 mice in Mφ medium in Teflon Pots for 7 days. 5 × 10^6^ mature CD45.1^+^ BMMφ were injected intravenously into wild-type CD45.2^+^ C57BL/6 mice on days 3 and 4 following UUO (total cells injected = 15 × 10^6^). The obstructed left kidney, sham controls and draining lymph nodes were harvested on day 7 and either digested for flow cytometric studies or snap frozen for immunofluorescent staining. In some experiments, liver and spleen were harvested and enzymatically dissociated prior to flow cytometric studies.

### 2.12 Detection of CD45.1^+^ cells in the lymph node draining the kidney

Frozen sections of the draining lymph nodes were air dried for 30 min. and fixed in ice-cold acetone for 5 min. Tissue was blocked for 1 h with M.O.M.^™^ Ig Blocking Reagent (Vector) diluted in PBS, washed in PBS, incubated for 5 min in the M.O.M.^™^ Diluent (Vector) before incubating with mouse anti-mouse CD45.1 (Clone A20 Biolegend; 1:250 dilution in MOM diluent) for 1 h at room temperature. Slides were washed, incubated in goat anti-mouse Alexa Fluor 488 (Invitrogen, 1:300 in Dako antibody diluent) for 30 min., washed and mounted with Vetashield medium (plus DAPI for nuclear staining; Vector). Images were captured using a Zeiss Axioskop 2mot + microscope (Zeiss).

### 2.13 Statistical analysis

Data were analysed with Prism software (GraphPad). All values are expressed as mean ± SEM. Unpaired Student’s *t*-test or ANOVA (Tukey post-hoc test) were used for analysis. P-values < 0.05 denote statistical significance, *p < 0.05, **p < 0.01, ***p < 0.005.

## 3 Results

### 3.1 GM-CSF treatment of macrophages induces DC characteristics

**Fig. 1:**
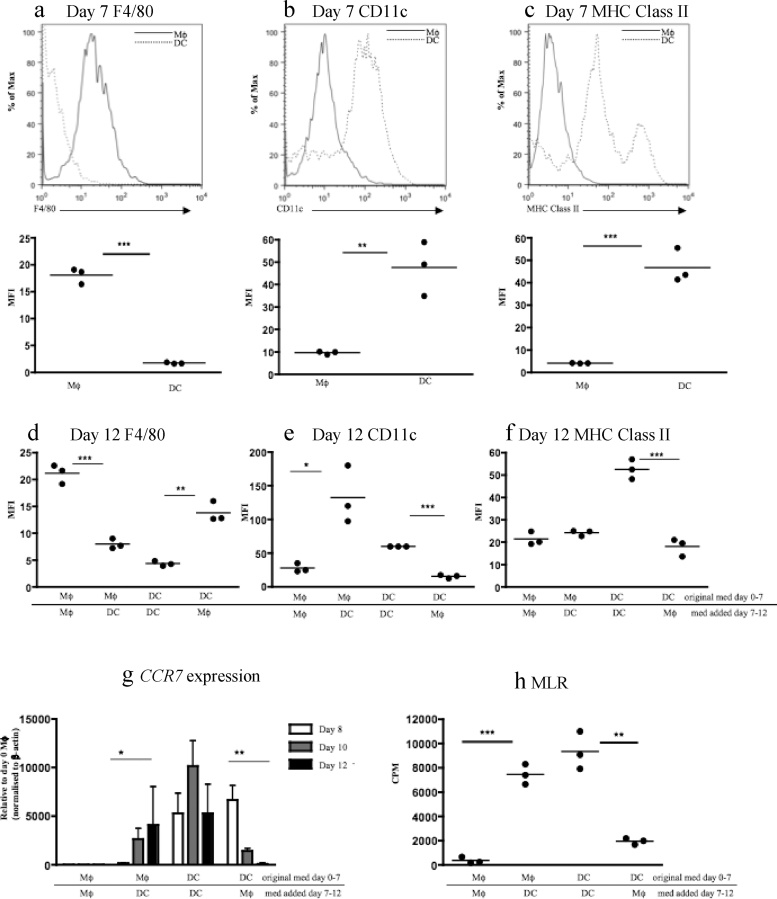
**Macrophages can be induced to adopt properties characteristic of dendritic cells and** vice versa. Bone marrow cells were grown in macrophage (Mφ) or dendritic cell (DC) media for 7 days prior to assessment of cell surface expression of F4/80 (**a**), CD11c (**b**) and MHC Class II (**c**) by flow cytometry. The level of expression is expressed as mean fluorescent intensity (MFI). Day 7 bone marrow-derived Mφ and DCs were then washed and incubated for a further 5 days in DC medium or Mφ medium respectively. Control Mφ and DCs underwent further culture in Mφ or DC medium respectively. At day 12, cells were recovered and the expression of F4/80 (**d**), CD11c (**e**) and MHC Class II (**f**) was determined. Again, day 7 bone marrow-derived Mφ and DCs were either switched to DC or Mφ medium for a further 5 days. Now cells were recovered at days 8, 10 and 12 and real-time PCR undertaken for *CCR7* mRNA expression (**g**). Day 12 cells from control Mφ or DC cultures or ‘medium switched’ cultures from C57BL/6 mice were replated with splenocytes from BALB/c animals for 48 h. 1 mCi of [3 H]-TdR was added to each well and plates incubated overnight before harvesting and counting using a liquid scintillation counter to measure T cell proliferation. Data expressed as counts per minute (cpm) (**h**). Results are representative of multiple experiments (>3). * p < 0.05, ** p < 0.01, ***p < 0.

Initial experiments phenotyped BMMφ or BMDCs generated by standard C57BL/6 bone marrow culture for 7 days in medium conditioned with either M-CSF (Mφ medium) or GM-CSF (DC medium; Supplementary Fig. 1a). Flow cytometry indicated that day 7 BMMφ were F4/80^Hi^CD11c^Low^Class II^Low^ whilst day 7 BMDCs were F4/80^Low^CD11c^Hi^Class II^Hi^ (Fig. 1a–c).

Day 7 BMMφ were then cultured for a further 5 days in either Mφ medium (M-CSF) or DC medium (GM-CSF; Supplementary Fig. 1b) and phenotyped by flow cytometry. Culture of F4/80^Hi^CD11c^Low^Class II^Low^ BMMφ in DC medium resulted in reduced F4/80 expression and increased CD11c expression with no change in Class II expression (Fig. 1d–f). Analysis of primary peritoneal Mφ revealed a similar pattern following 5 days treatment with DC medium (Supplementary Fig. 2). Day 7 BMDC were also cultured for a further 5 days in either Mφ medium or DC medium and phenotyped by flow cytometry (Supplementary Fig. 1b). Culture of F4/80^Low^CD11c^Hi^Class II^Hi^ BMDCs in Mφ medium resulted in increased F4/80 expression and reduced CD11c and MHC Class II expression (Fig. 1d–f). Results similar to those above were obtained using recombinant M-CSF and GM-CSF (Supplementary Fig. 3), and using cells derived from FVBN/j bone marrow (data not shown) suggesting that the modulatory effects were directly driven by the growth/differentiation factors and were not unique to cells derived from C57/BL6 mice.

Expression of *CCR7* mRNA was high in d7 BMDCs and absent in d7 BMMφ (data not shown). Realtime PCR for *CCR7* mRNA expression in BMMφ was performed at days 8, 10 and 12 following the change of medium at day 7 (Mφ→DC medium) and demonstrated a progressive increase in *CCR7* mRNA expression by BMMφ cultured in DC medium reaching statistical significance by day 12 (Fig. 1g). In contrast, a progressive loss of *CCR7* expression was evident in BMDCs cultured in Mφ medium (Fig. 1g). As expected, control day 7 BMMφ cultured in Mφ medium for a further 5 days did not upregulate *CCR7* mRNA expression whilst BMDCs cultured in DC medium for a further 5 days exhibited an ongoing high level of *CCR7* mRNA expression (Fig. 1g).

CCR7 expression is a feature of DCs as it facilitates the emigration of DCs from tissues to lymph nodes. As well as this, DCs also have the capacity to stimulate naïve allogeneic T cells. We therefore tested whether the culture of BMMφ in DC medium induced the ability to stimulate naïve allogeneic T cells. As shown in Fig. 1h, day 7 BMMφ cultured in DC medium for 5 days acquired the ability to stimulate naïve allogeneic T cells in a mixed leukocyte reaction (MLR). In contrast, day 7 BMDCs cultured in Mφ medium for 5 days exhibited a significant reduction in the ability to drive T cell proliferation (Fig. 1h). As expected, BMMφ or BMDCs maintained throughout in Mφ or DC medium respectively for 12 days retained their characteristic phenotypic markers and functional properties (Fig. 1).

### 3.2 GM-CSF treatment reduces the phagocytic capacity of macrophages

**Fig. 2:**
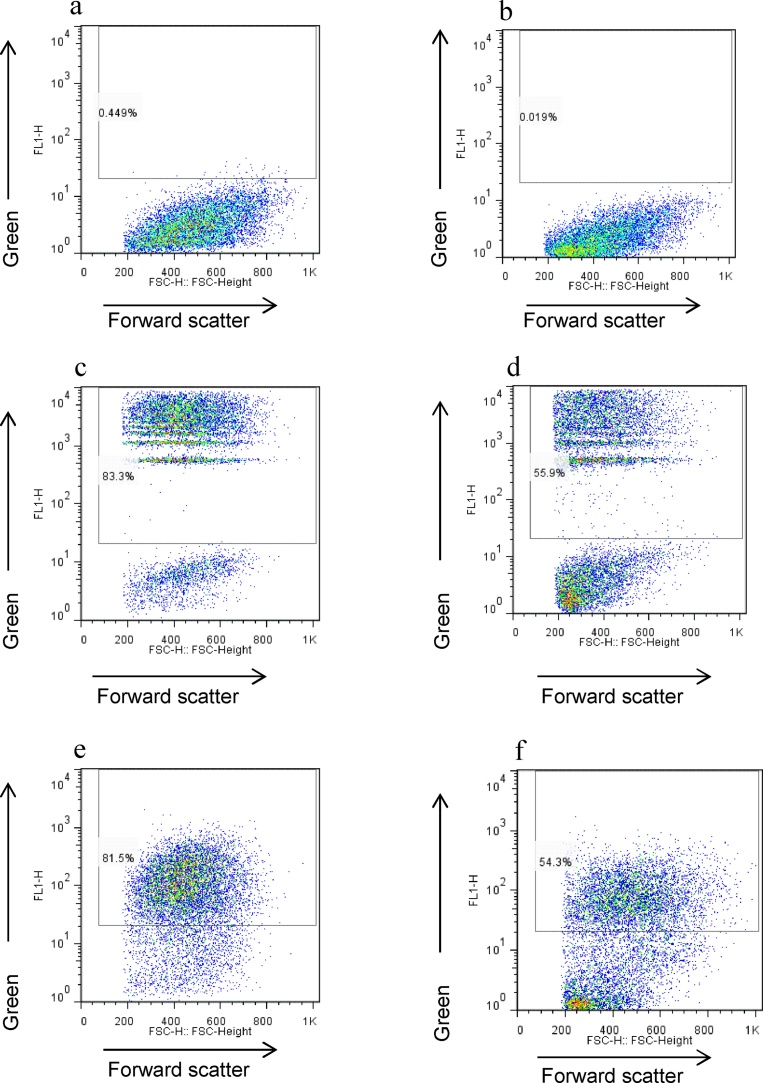

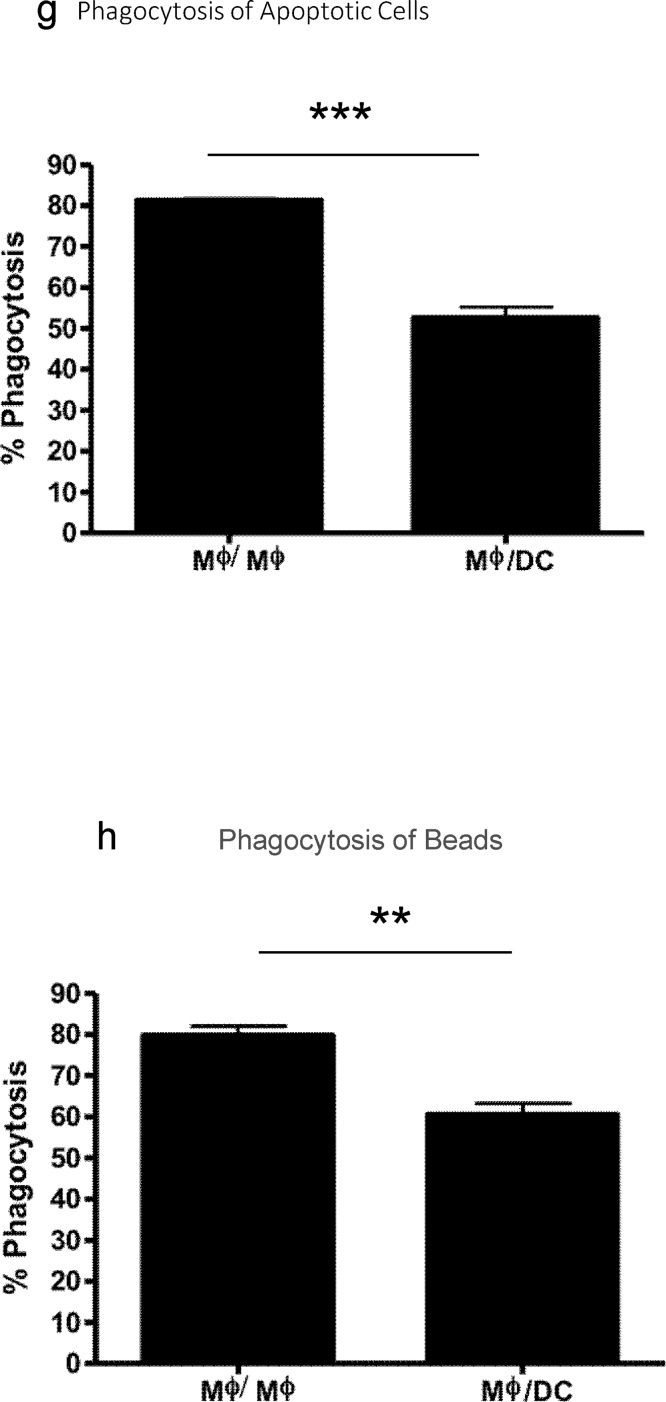
**Culture in DC medium reduces macrophage phagocytosis of apoptotic cells and beads.** Day 7 bone marrow-derived Mφ were incubated for 5 days in macrophage (Mφ) or dendritic cell (DC) medium (n = 3 mice). Cells were then incubated with CellTracker Green labeled apoptotic murine thymocytes or 3 μm Fluoresbrite YG microsphere beads for 60 min. Cells were vigorously washed and detached from the plates for flow cytometric assessment of the proportion of cells that had phagocytosed apoptotic cells or beads. Representative flow cytometry dot plots of Mφ cultured in Mφ medium (cells alone (**a**), following incubation with beads (**c**) or apoptotic cells (**e**)) or DC medium (cells alone (**b**), following incubation with beads (**d**) or apoptotic cells (**f**). Quantification of the proportion of cells exhibiting phagocytosis demonstrates that Mφ cultured in Mφ medium (Mφ/Mφ) remain highly phagocytic but exhibit reduced phagocytic capacity following incubation in DC medium (Mφ/DC) (**g** & **h**). ** p < 0.01, ***p < 0.001 (For interpretation of the references to colour in this figure legend, the reader is referred to the web version of this article).

The phagocytic ingestion of apoptotic cells and cell debris is a key feature of both homeostatic and inflammatory Mφ, and plays an important role in tissue development, inflammation and healing (Elliott et al., 2017). We therefore used a flow cytometric assay to examine whether exposure to DC medium modulated Mφ phagocytosis of CMgreen labelled apoptotic cells and fluorescent beads (Fig. 2a–h). BMMφ cultured in Mφ medium for 12 days were markedly phagocytic with ∼80% of cells ingesting apoptotic cells or beads (Fig. 2a,c&e). The culture of BMMφ in DC medium for 5 days significantly reduced the level of phagocytosis of both apoptotic cells and fluorescent beads (Fig. 2b,d,f–h).

### 3.3 Modulation of bone marrow-derived macrophages and dendritic cell phenotype result from whole cell population changes

**Fig. 3:**
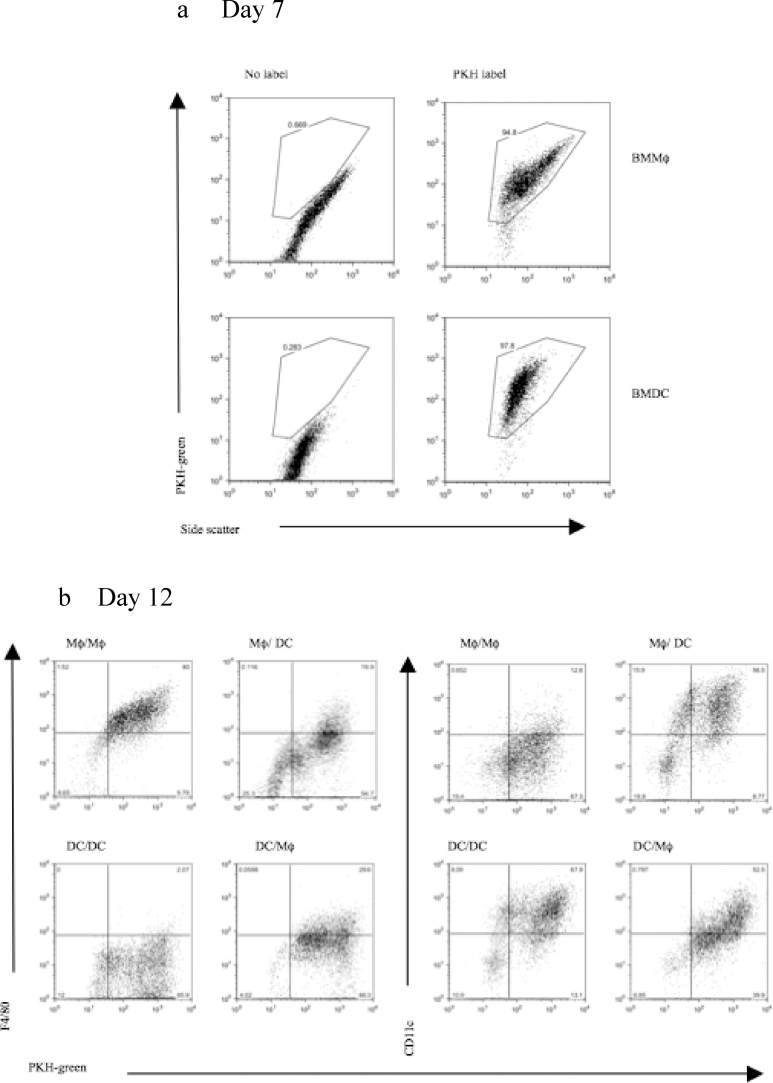
**The phenotypic shifts of bone marrow-derived macrophages and dendritic cells are due to whole cell population changes and not to an outgrowth of progenitor cells.** Day 7 bone marrow derived macrophages (BMMφ) or dendritic cells (BMDC) were labeled with PKH green. Flow cytometry was carried out to confirm that all cells were labeled with PKH dye (**a**). The labeled Mφ and DCs were then replated and cultured in either the same medium (control Mφ/Mφ and DC/DC) or switched to the opposite medium (Mφ/DC and DC/Mφ) for a further 5 days. At day 12 cells were analysed by flow cytometry to assess the expression of F4/80 and CD11c (y-axis) and the presence of PKH green (x-axis) (**b**).

It was important to determine whether the modulatory effects of the 5-day culture period in either Mφ or DC medium resulted from the proliferation and outgrowth of a progenitor cell population present within the initial day 7 BMMφ or BMDC population with the associated death of established mature BMMφ or BMDC from day 7 onwards. We therefore undertook a series of cell labeling studies to enable cells to be tracked over time. BMMφ or BMDCs were labeled with PKH67 green fluorescent dye at day 7 prior to medium switching or continuation for a further 5 days. 95–98% of d7 BMMφ and BMDCs were labeled with PKH67 green (Fig. 3a) with cells retaining the dye over the ensuing 5 days (Fig. 3b). Flow cytometry dot plots indicated that the entire population of PKH labeled d7 BMMφ exhibited decreased F4/80 expression and increased CD11c expression following culture in DC medium with the opposite results for BMDCs cultured in Mφ medium (Fig. 3b). Thus, the induced phenotypic changes in BMMφ and BMDCs cultured in DC medium or Mφ medium respectively resulted from whole population shifts and not the rapid expansion of progenitor populations associated with the death of mature BMMφ and BMDCs from day 7 onwards.

### 3.4 Concurrent exposure to both GM-CSF and M-CSF modulates the phenotype of macrophages and dendritic cells

**Fig. 4:**
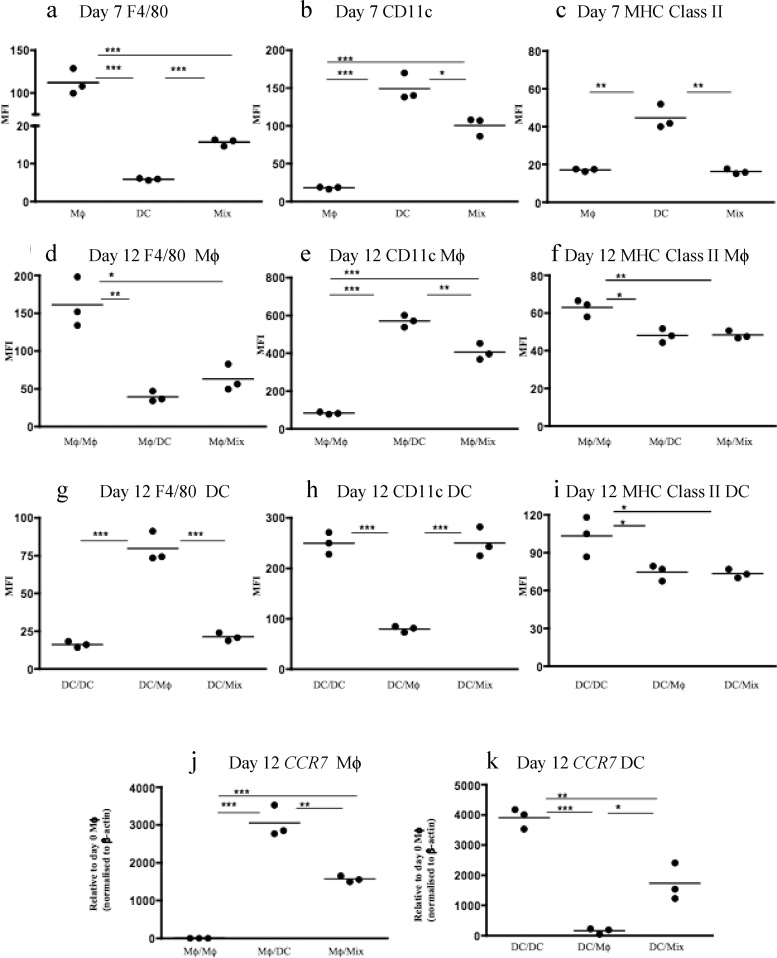
**Exposure to a 50:50 mix of macrophage and dendritic cell medium modulates the phenotype of macrophages and dendritic cells**. Bone marrow cells were grown in either macrophage (Mφ) medium, DC medium or a 50:50 mix of Mφ and DC media (Mφ, DC or mix) for 7 days before prior to analysis of cell expression of F4/80 (**a**), CD11c (**b**) and MHC Class II expression (**c**) by flow cytometry. The level of expression is expressed as mean fluorescent intensity (MFI). In further experiments, day 7 bone marrow-derived Mφ were washed and incubated for a further 5 days in either Mφ medium (Mφ/Mφ), DC medium (Mφ/DC) or the 50:50 mix (Mφ/mix). Similarly, day 7 bone marrow-derived DCs were washed and incubated for a further 5 days in either DC medium (DC/DC), Mφ medium (DC/Mφ) or the 50:50 mix (DC/mix). At day 12, cells were recovered and the expression of F4/80 (**d, g**), CD11c (**e, h**) and MHC Class II (**f, i**) was determined by flow cytometry whilst real-time PCR was used to determine *CCR7* mRNA expression (**j, k**). Results are representative of multiple experiments (>3). * p < 0.05, ** p < 0.01, ***p < 0.001.

The above experiments indicated that the *in vitro* phenotype of mature BMMφ and BMDCs can be modulated by the discrete and sole exposure to GM-CSF or M-CSF respectively. Although conditions *in vitro* may be carefully controlled, monocytes recruited from the blood *in vivo* are likely to be exposed to both M-CSF and GM-CSF during renal inflammation (Matsuda et al., 1996), as many renal cells may produce M-CSF (Isbel et al., 2001a; Isbel et al., 2001b) and GM-CSF (Greiber et al., 2002; Dudas et al., 2011). We hypothesized that the simultaneous exposure to both M-CSF and GM-CSF would also act to modulate cell phenotype and we therefore cultured bone marrow cells from the outset in either Mφ medium, DC medium or a 50:50 mix of Mφ/DC media in an attempt to simulate the exposure of monocytes to multiple signals that might be found in the tissue environment. Cells that had been exposed to the 50:50 mix Mφ/DC media for 7 days exhibited a phenotype somewhat intermediate between BMMφ and BMDCs being F4/80^Med^CD11c^Med^Class II^Low^ (Fig. 4a–c).

We then assessed the effect of simultaneous exposure to both M-CSF and GM-CSF upon d7 mature BMMφ and BMDC by culturing them in 50:50 mix of Mφ/DC media for 5 days prior to phenotyping in an attempt to simulate the exposure of mature tissue Mφ and DCs to multiple signals that might be found in inflamed tissues. Intriguingly, BMMφ cultured for 5 days in the 50:50 mix of Mφ/DC media exhibited reduced F4/80, increased CD11c, reduced MHC Class II and increased *CCR7* mRNA expression compared to control BMMφ (Fig. 4d-f&j). In contrast, d7 BMDCs exposed to the 50:50 mix of Mφ/DC media for 5 days exhibited reduced expression of MHC Class II and *CCR7* mRNA and comparable F4/80 and CD11c expression compared to control BMDCs (Fig. 4h-i&k). The effect of such combinatorial stimuli upon phenotype is complicated by the fact that GM-CSF can downregulate the expression of colony-stimulating factor-1 receptor (CSF1R) that binds M-CSF and thus indirectly inhibit M-CSF signaling (Panterne et al., 1996). Indeed, we noted that culture of BMMφ with DC medium for 5 days reduced cell surface expression of CSF1R by ∼60% (data not shown).

### 3.5 The obstructed murine kidney expresses both M-CSF and GM-CSF

**Fig. 5:**
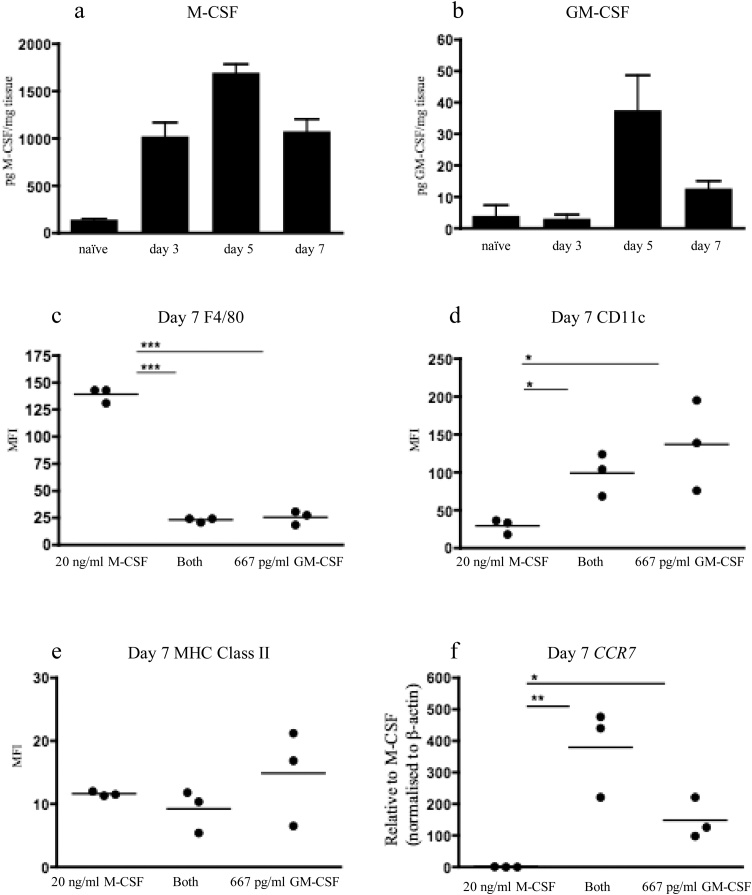
**The obstructed kidney expresses both M-CSF and GM-CSF with modulation of macrophage and dendritic cell phenotype by recombinant M-CSF and GM-CSF.** C57BL/6 mice underwent unilateral ureteric obstruction and kidneys were removed at days 3, 5 and 7 for protein extraction. The concentrations of M-CSF (**a**) and GM-CSF (**b**) were determined by ELISA. Bone marrow cells were cultured for 7 days in either recombinant M-CSF (20 ng/ml), GM-CSF (667 pg/ml) or a 30:1 mix (both) of M-CSF and GM-CSF (20 ng/ml M-CSF: 667 pg/ml GM-CSF). At day 7, the expression of F4/80 (**c**), CD11c (**d**) and MHC Class II (**e**) was determined by flow cytometry whilst real-time PCR was used to determine *CCR7* mRNA expression (**f**). The level of expression is expressed as mean fluorescent intensity (MFI). Results are representative of multiple experiments. * p < 0.05, ** p < 0.01, ***p < 0.001.

Since the phenotype of BMMφ and BMDCs is modulated by the ambient M-CSF and GM-CSF levels *in vitro*, we determined the M-CSF and GM-CSF protein levels by ELISA of kidney homogenates following UUO. M-CSF and GM-CSF protein levels increased significantly by day 5 following UUO (Fig. 5a,b). The M-CSF/GM-CSF ratio following UUO varied over multiple experiments (Fig. 5a, b and data not shown) with a mean peak M-CSF/GM-CSF ratio of 30:1. Thus, although the obstructed kidney is a M-CSF dominant microenvironment, recruited monocytes would be exposed to low levels of GM-CSF.

### 3.6 Exposure to a 30:1 mix of recombinant M-CSF/GM-CSF modulates macrophage phenotype

Bone marrow cells were cultured for 7 days in either recombinant M-CSF (20 ng/ml), GM-CSF (667 pg/ml) or a 30:1 M-CSF/GM-CSF mix (Both; 20 ng/ml M-CSF & 667 pg/ml GM-CSF). Cells cultured with M-CSF generated F4/80^Hi^CD11c^Low^ cells whilst both GM-CSF and the M-CSF/GM-CSF mix generated F4/80^Low^CD11c^Hi^ cells (Fig. 5c, d). MHC Class II expression was low in all cells (Fig. 5e). The presence of GM-CSF alone or with M-CSF induced significant expression of *CCR7* mRNA (Fig. 5f). Thus, some conventional characteristics of DCs can be induced *in vitro* even when relatively small amounts of GM-CSF are present.

### 3.7 The phenotype of bone marrow-derived macrophages is modified following adoptive transfer to mice with ureteric obstruction

**Fig. 6:**
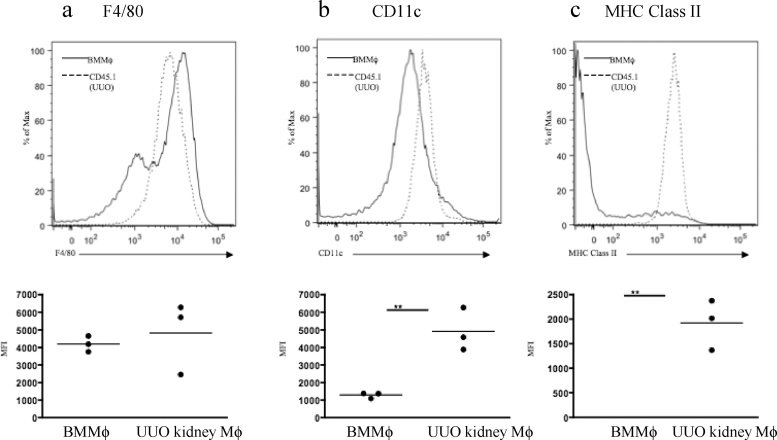
**The phenotype of bone marrow-derived macrophages is modified following adoptive transfer to mice with ureteric obstruction.** 15 × 10^6^ mature day 7 bone marrow-derived macrophages (BMMφ) generated from CD45.1^+^ C57BL/6 mice were intravenously administered to CD45.2^+^ C57BL/6 mice (WT C57BL/6) in 3 separate injections 3–4 days following unilateral ureteric obstruction (UUO). Whole kidneys were recovered and enzymatically dissociated for flow cytometric analysis at day 7 following UUO. Flow cytometry for F4/80 (**a**), CD11c (**b**) or MHC Class II (**c**) was undertaken on day 7 BMMφ on the day of injection and the CD45.1 cells from the digested kidneys. Representative histograms are shown (continuous line - day 7 injected BMMφ, dashed line - CD45.1 cells from obstructed kidney) whilst the level of expression is expressed as mean fluorescent intensity (MFI). ** p < 0.01.

**Fig. 7:**
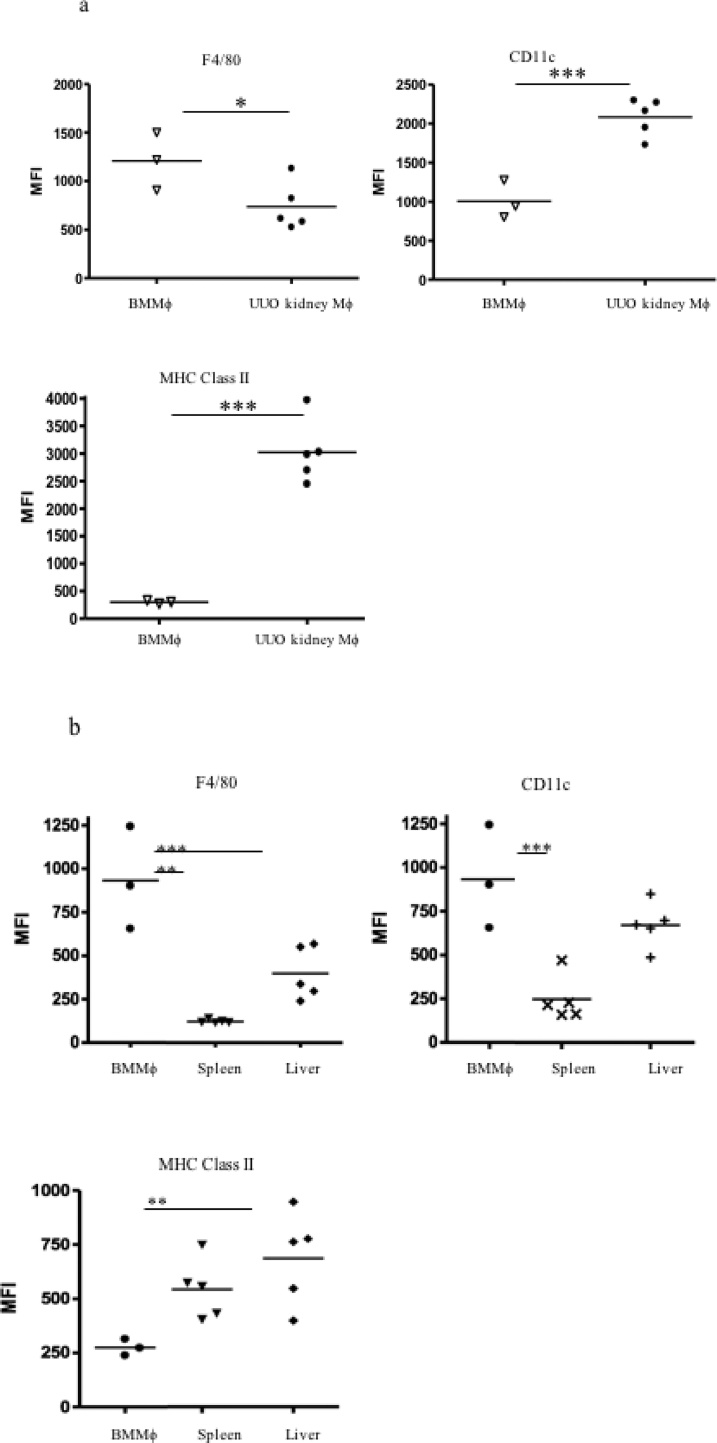
**The phenotype of adoptively transferred bone marrow-derived macrophages localizing to multiple organs.** Day 7 mature bone marrow-derived macrophages (BMMφ) generated from CD45.1^+^ C57Bl/6 mice were intravenously administered to CD45.2^+^ animals (WT C57BL/6) 3–4 days after unilateral ureteric obstruction (UUO) had been performed (total cells injected ∼15 × 10^6^) in order to track the fate of the injected BMMφ. The obstructed kidneys, liver, spleen and draining lymph node were recovered at day 7 and enzymatically dissociated for flow cytometric analysis of CD45.1+ cells for expression of F4/80, CD11c, MHC Class II and CD86 (the level of expression is quantified as mean fluorescent intensity [MFI]). CD45.1+ cells that localized to the obstructed kidney exhibited a reduction in F4/80, increased CD11c and a striking increase in MHC Class II expression with no change in CD86 expression (**a**). This phenotype was not evident in CD45.1+ cells that localized to the spleen (↓F4/80, CD11c & CD86) or liver (↓F4/80 & CD86, ↑ MHC Class II) (**b**). ** p < 0.01, ***p < 0.001.

**Fig. 8:**
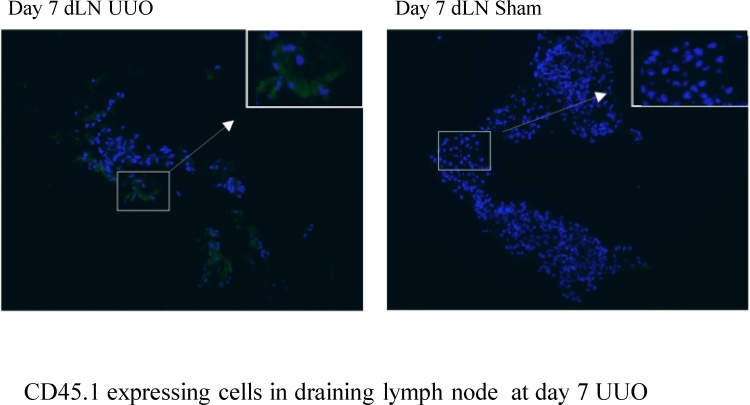
**Adoptively transferred bone marrow-derived macrophages localize to the draining renal lymph node in mice with ureteric obstruction.** 15 × 10^6^ mature day 7 bone marrow-derived macrophages (BMMφ) generated from CD45.1^+^ C57BL/6 mice were intravenously administered to CD45.2^+^ C57BL/6 mice (WT C57BL/6) in 3 separate injections 3–4 days following unilateral ureteric obstruction (UUO). The lymph nodes draining the left kidney following UUO or sham surgery were removed and CD45.1 expression detected by immunofluorescent microscopy (CD45.1 - Green, DAPI - blue) (For interpretation of the references to colour in this figure legend, the reader is referred to the web version of this article).

We then asked whether such phenotypic changes might be induced in exogenously administered BMMφ following recruitment to the obstructed kidney *in vivo.* A total of 15 × 10^6^ CD45.1^+^ BMMφ was administered to CD45.2^+^ mice (3 separate intravenous injections of 5 × 10^6^ CD45.1^+^ BMMφ) on days 3 and 4 following UUO. Flow cytometric analysis of digested kidneys indicated the presence of CD45.1^+^ cells at day 7 with very few CD45.1^+^ cells evident at day 10 suggesting recruitment of administered CD45.1^+^ BMMφ at day 7 and either death or egress from the kidney at d10 (Supplementary Fig. 4). Compared to the CD45.1^+^ BMMφ administered at d 3-4, the CD45.1^+^ cells present in dissociated obstructed kidneys at day 7 expressed comparable F4/80 expression, increased CD11c expression and markedly increased MHC Class II expression (Fig. 6a–c). Additional experiments were performed to examine the phenotype of CD45.1^+^ BMMφ that had localized to other organs such as liver and spleen. In these studies, the CD45.1^+^ cells present in the dissociated UUO kidneys at day 7 expressed reduced F4/80 expression, increased CD11c and MHC Class II expression compared to the CD45.1^+^ BMMφ administered at d 3-4 (Fig. 7a). Some modulation of phenotype was evident in other organs but the renal phenotype was not recapitulated in CD45.1^+^ cells isolated from liver or spleen (Fig. 7b). Since our *in vitro* data indicated prominent upregulation of *CCR7* mRNA expression by exposure of BMMφ to GM-CSF, we examined the draining renal lymph nodes for the presence of CD45.1^+^ cells that would suggest BMMφ egress from the kidney. Immunofluorescent staining of the draining left renal hilar lymph node of obstructed kidneys revealed the presence of CD45.1^+^ cells (Fig. 8) with no CD45.1^+^ cells found in lymph nodes of controls that underwent sham surgery 3 days before the intravenous administration of equal numbers of CD45.1^+^ BMMφ. This is supported by evidence that *CCR7* expression is increased in CD11b + mononuclear phagocytes in the kidney after UUO (Supplementary Fig. 5).

## 4 Discussion

Mφ and DCs are extremely heterogeneous and display significant plasticity due to stimuli present in their local environment (Hochheiser et al., 2011; Mosser and Edwards, 2008; Davies et al., 2013). This work supports the concept that colony stimulating factors play a crucial role in mononuclear phagocyte phenotype in the kidney after injury (Isbel et al., 2001a; Isbel et al., 2001b; Zhang et al., 2012; Wang et al., 2015; Huen et al., 2015), and emphasizes a key role for GM-CSF.

Tubulointerstitial mononuclear phagocytes are part of the intricate system that surveys against injury and infection (Weisheit et al., 2015), and contributes to organ homeostasis and tissue repair (Wang and Harris, 2011; Ferenbach et al., 2010; Rogers et al., 2014). These myeloid cells are crucial in the recovery of kidney function after injury, and can dictate the balance between successful regeneration and progressive fibrosis (Rogers et al., 2014). Mφ are very responsive to their environment and exhibit different states of maturity, activation, polarization and plasticity *in vivo* (Mosser and Edwards, 2008). For example, Mφ may adopt a pro-inflammatory M1 or an anti-inflammatory wound healing M2 phenotype (Nelson et al., 2012). M1 Mφ may evolve into M2 Mφ following acute kidney injury (Lee et al., 2011), whilst M2 Mφ exhibit M1 features following LPS stimulation (Mylonas et al., 2009). Whereas pro-inflammatory Mφ contribute to the initial kidney damage, a M2 Mφ phenotype can promote normal renal repair. DCs are closely related to Mφ, but are considered professional APCs, whose main role is the activation and regulation of T cells. CCR7 is an important marker of DCs as it directs their emigration from tissues towards the chemokines CCL19/21 in lymphatic tissue (Hirao et al., 2000; Yu et al., 2008; Jakubzick et al., 2006).

Both M-CSF and GM-CSF were elevated during UUO. Therefore, mononuclear phagocytes present in that environment are likely to be exposed to both M-CSF and GM-CSF such that cells grown in either M-CSF or GM-CSF are unlikely to have a counterpart *in vivo*. There is an ongoing debate regarding the classification of mononuclear phagocyte as either a Mφ or DC (Hume, 2008), and recent work indicates that both these cells in the kidney may exhibit features previously ascribed to either one or the other, e.g. expressing both F4/80 and CD11c (Cao et al., 2015; Kruger et al., 2004; Bethunaickan et al., 2011). Thus, a somewhat ‘hybrid’ cell, similar to the cells identified in our studies, may well be present *in vivo*. Indeed, in the steady state, renal mononuclear phagocytes share both Mφ and DC characteristics, appear to serve as sentinels for the immune response, and play anti-inflammatory and tissue-reparative roles (Kawakami et al., 2013). CD11c + subpopulations of mononuclear phagocytes from the kidney, preferentially induce regulatory T cell differentiation upon presentation to naïve T cells, implicating a role in immunological tolerance (Kawakami et al., 2013).

During the inflammatory model of unilateral ureteric obstruction (UUO), used in this study, the replacement of functional kidney tissue with scarred tissue has been found to be highly dependent on Mφ, as depletion of these cells with clodronate greatly reduces fibrosis (Viehmann et al., 2018). CD11c + mononuclear phagocytes, historically described as ‘DCs’, have been found to play no role in the fibrotic response during UUO but display enhanced antigen presentation to T cells. Studies indicate that these antigen presenting CD11c + cells actually help reduce UUO-induced inflammation and fibrosis (Viehmann et al., 2018), possibly through preferential promotion of regulatory T cells in the lymphatics (Kawakami et al., 2013), although this requires further investigation.

We found that M-CSF levels were greatly elevated by day 3 and peaked at day 5 post-UUO. In tissue, production of M-CSF induces monocyte recruitment from the blood and proliferation and survival of tissue-resident Mφ. Indeed, previous studies in the kidney have found that after renal injury, M-CSF produced specifically by tubular epithelial cells is important for the proliferation of resident Mφ, and monocyte recruitment (Zhang et al., 2012; Wang et al., 2015). In agreement with this, we noted rising levels of M-CSF when Mφ/DC numbers were increasing in the UUO kidney. We, and others (Smith et al., 2013), have found that M-CSF also promotes phagocytosis. These actions of M-CSF in an *in vivo* context likely contributes to apoptotic cell clearance and M1 to M2 phenotypic switching, thus promoting the resolution of inflammation (Reviewed in Savill et al., 2002 and Elliott et al., 2017). In support of this, Wang et al demonstrated that the absence of tubular cell-derived M-CSF led to reduced phenotype switching of Mφ from an M1 to a the M2 phenotype that is essential for effective renal repair (Wang et al., 2015). Therefore, M-CSF is crucial for an increase in Mφ numbers early post-injury, augmentation of phagocytosis and skewing towards a reparative M2 phenotype at later time-points.

GM-CSF is present at low levels in the steady state, but is induced by inflammation, driving such ‘DC’ functions as chemotaxis (Shiomi and Usui, 2015) and antigen presentation (Morrissey et al., 1987). Although, reports suggest that GM-CSF drives M1 activation *in vitro* (Brocheriou et al., 2011; Lacey et al., 2012; Fleetwood et al., 2007), it has recently been found to be critically important for M2 Mφ activation during kidney injury (Huen et al., 2015). The current study revealed that the peak protein expression level of GM-CSF post-UUO (day 5), was less than 4% of the expression level of M-CSF. However, our studies indicated that relatively low levels GM-CSF exerted a dominant effect over M-CSF on cellular phenotype *in vitro*. Mφ, initially cultured in M-CSF and then exposed to DC medium, displayed decreased Mφ markers and reduced phagocytic activity. The strong induction of *CCR7* mRNA in BMMφ by GM-CSF *in vitro*, coupled with detecting adoptively transferred BMMφ in the draining renal lymph node *in vivo* suggests that GM-CSF drives CCR7 up-regulation *in vivo* in Mφ, which promotes egress from the kidney (Lan et al., 1993), as has been described for foamy Mφ in atheroma (Trogan et al., 2006). This was supported by our evidence that *CCR7* expression is significantly increased in CD11b + mononuclear phagocytes in the kidney after UUO.

GM-CSF may reduce the responsiveness of mononuclear phagocytes to M-CSF. Cells differentiated in GM-CSF express splice variants of CSF-1R (the receptor for M-CSF) that may encode a soluble decoy receptor (Hume, 2008). GM-CSF can also promote the cleavage of cell surface CSF-1R (Hiasa et al., 2009). This inevitably leads to disruption of M-CSF signalling, resulting in GM-CSF exerting a dominant effect, as was evident in this study at very low levels of GM-CSF relative to M-CSF.

**Fig. 9:**
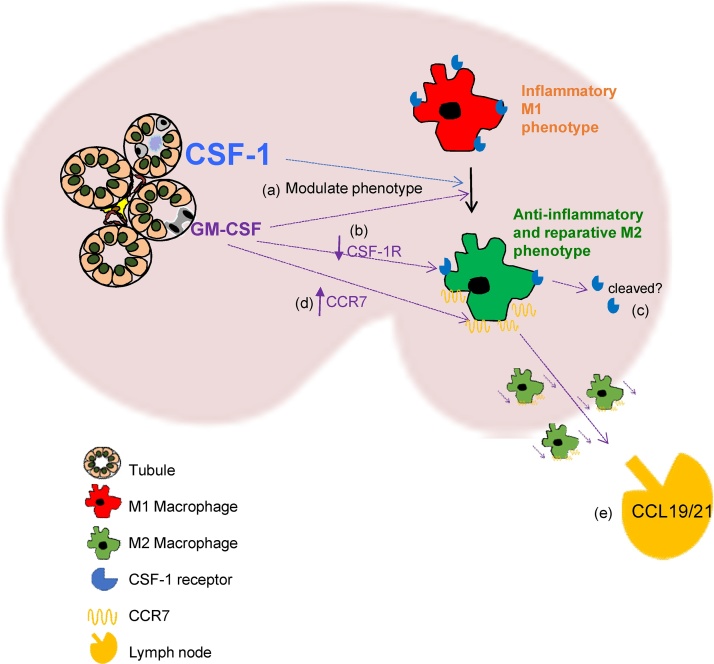
**Schematic diagram of the potential effects of M-CSF and GM-CSF upon macrophages in the obstructed kidney.** M-CSF contributes to the switch of M1 inflammatory Mφ to a reparative M2 phenotype (**a**). GM-CSF, despite being expressed at lower levels compared to M-CSF, also contributes to the M1 to M2 Mφ phenotypic shift (**a**). GM-CSF causes a reduction of CSF1-R expression (**b**) possibly through cleavage of the CSF1-R receptor (**c**).^47^ GM-CSF induces the upregulation of the chemokine receptor CCR7 in Mφ (**d**) thereby promoting chemotaxis of Mφ to the chemokines CCL19 and CCL21 expressed by lymphatic vessels (**e**). Thus may, GM-CSF promote the egress of CCR7^+^ cells from the inflamed kidney to draining lymph nodes.

Therefore, consistent with our current *in vitro* and *in vivo* findings and the work of others (Zhang et al., 2012; Wang et al., 2015; Huen et al., 2015; Savill et al., 2002; Elliott et al., 2017; Hiasa et al., 2009), we propose the following roles of M- and GM-CSF following UUO- M-CSF expression increases in tubules early after injury (by day 3) and promotes the recruitment and proliferation of mononuclear phagocytes, as part of the initial inflammatory response. By day 5 (Fig. 9) M-CSF contributes to the switching of these inflammatory M1 Mφ to a wound healing M2 phenotype. GM-CSF expression has also increased and drives an M1 to M2 Mφ phenotypic shift (Fig. 9), promoting the resolution of inflammation. We propose that GM-CSF expression also reduces the responsiveness of mononuclear phagocytes to M-CSF, thus preventing further recruitment of monocytes/proliferation of resident Mφ. GM-CSF also drives the upregulation of CCR7, which promotes the egress of these cells from the inflamed kidney (Fig. 9), which may assist in the dampening of UUO-induced inflammation and fibrosis by removing inflammatory cells and/or potentially stimulating regulatory T cells.

## 4.1 Conclusion

In conclusion, this study reinforces the concept that Mφ and myeloid DCs represent points on a continuum within the mononuclear phagocyte system (Hume et al., 2002, Hume, 2008). We propose that GM-CSF plays a key role in regulating mononuclear phagocyte plasticity during renal inflammation, even when present in relatively small levels, and exerts actions that contributes to the resolution of inflammation and promotion of renal repair.

## Declarations of interest

None. The authors disclose no competing financial interests.

## Author contributions

JH and KJM conceived, designed the experiments and wrote the manuscript. KJM, JA, TAS, EEH and JAR carried out experimental procedures. DAF, DCK and JS contributed towards the manuscript.

## Competing financial interests

The authors disclose no competing financial interests.
