# Supplementary material for: Granulocyte macrophage-colony stimulating factor: A key modulator of renal mononuclear phagocyte plasticity
Source: Immunobiology. 2019 Jan;224(1):60–74. doi: 10.1016/j.imbio.2018.10.007 (PMC6401212; doi:10.1016/j.imbio.2018.10.007)
Supplement: Supplementary file 1 [file mmc1.docx]

Supplementary Material
